# Real-Time Small Drones Detection Based on Pruned YOLOv4

**DOI:** 10.3390/s21103374

**Published:** 2021-05-12

**Authors:** Hansen Liu, Kuangang Fan, Qinghua Ouyang, Na Li

**Affiliations:** 1 School of Mechanical and Electrical Engineering, Jiangxi University of Science and Technology, Ganzhou 341000, China; hansenliumail@163.com; 2 Institute of Permanent Maglev and Railway Technology, Jiangxi University of Science and Technology, Ganzhou 341000, China; ouyang15770664356@163.com (Q.O.); 15607076773@163.com (N.L.); 3 School of Electrical Engineering and Automation, Jiangxi University of Science and Technology, Ganzhou 341000, China

**Keywords:** anti-drone, YOLOv4, pruned deep neural network, small object augmentation

## Abstract

To address the threat of drones intruding into high-security areas, the real-time detection of drones is urgently required to protect these areas. There are two main difficulties in real-time detection of drones. One of them is that the drones move quickly, which leads to requiring faster detectors. Another problem is that small drones are difficult to detect. In this paper, firstly, we achieve high detection accuracy by evaluating three state-of-the-art object detection methods: RetinaNet, FCOS, YOLOv3 and YOLOv4. Then, to address the first problem, we prune the convolutional channel and shortcut layer of YOLOv4 to develop thinner and shallower models. Furthermore, to improve the accuracy of small drone detection, we implement a special augmentation for small object detection by copying and pasting small drones. Experimental results verify that compared to YOLOv4, our pruned-YOLOv4 model, with 0.8 channel prune rate and 24 layers prune, achieves 90.5% mAP and its processing speed is increased by 60.4%. Additionally, after small object augmentation, the precision and recall of the pruned-YOLOv4 almost increases by 22.8% and 12.7%, respectively. Experiment results verify that our pruned-YOLOv4 is an effective and accurate approach for drone detection.

## 1. Introduction

Drones, also called unmanned aerial vehicles (UAVs), are small and remotely controlled aircraft that have experienced explosive growth and development in recent years. However, given the widespread use of amateur drones, an increasing number of public security threats and social problems have arisen. For example, commercial aircraft may be disturbed by drones when they appear in the same channel; drones may also invade no-fly zones or high-security areas [1,2,3].

Therefore, there is a significant need for deploying an anti-drone system that is able to detect drones at the time when they enter high-security areas. A radar that can analyze the micro-Doppler signatures is a traditional and effective tool for anti-drone systems [1,2]. In [4], frequency modulated continuous wave radars were used to detect mobile drones. However, it requires expensive devices to implement and may be inappropriate in crowded urban areas or those areas with complex background clutter because distinguishing drones from complex backgrounds is difficult at low altitudes [5,6]. Many studies use an acoustic signal to detect drones [7,8,9]. The acoustic signal captured by an acoustic uniform linear array (ULA) was used to estimate the direction of arrival (DOA) of a drone, and this method has achieved that the DOA absolute estimation error was no more than 6° [7]. Aspherical microphone array composed of 120 elements and a video camera was developed to estimate the 3D localization of UAVs using the DOA [10]. In addition to the acoustic-based method, a framework based on the received signal strength (RSS) of the radiofrequency signal was used to do both detection and localization [9]. However, acoustic-based detection is easily affected by environmental noise. Moreover, solutions that use machine learning or deep learning have elicited increasing attention due to the proliferation of artificial intelligence. 

Over the past few years, the threats posed by drones have been receiving considerable critical attention. Drone detection based on image processing has become increasingly popular among researchers in recent years. To address the problem of fast-moving drones, a drone detection method based on a single moving camera adopted a low rank-based model to obtain proposed objects; then, a convolutional neural network (CNN)–support vector machine (SVM) confirmed real drone objects [10]. Wang et al. presented a flying small target detection method over separated target images based on Gaussian Mixture Model [11]. Thus, we use an image-based detection method, which is simple and efficient. With the advent and huge increase of the application of deep neural network (DNN) in various areas, many problems that could not be solved before are currently being addressed [12,13]. Especially, the deep CNN (DCNN) is widely used in various tasks related to images, such as object detection, object tracking,, etc. A forest fire detection method based on CNN was developed in [14]. Benjdira used aerial images to accurately detect cars and count them in real time for traffic monitoring purposes by considering two CNNs: faster region-based CNN (RCNN) and You Only Look Once (YOLO) v3 [15]. In [16], Anderson proposed and evaluated the use of CNN-based methods combined with high spatial resolution RGB drone imagery for detecting law-protected tree species. In this previous work, the author compared three state-of-the-art CNNs: faster RCNN, RetinaNet and YOLOv3. A lot of research about anti-drone systems using deep learning and multi sensor information fusion have been discussed in [17]. 

Motivated by this, we use state-of-the-art CNN methods to achieve high drone detection accuracy. In this research, we evaluate four DCNNs, namely, RetinaNet, fully convolutional one-stage object detector (FCOS) [18], YOLOv3 and YOLOv4. However, the deployment of real-time detection drones is mostly constrained by two problems. One of them is that drones move quickly, which therefore requires faster detectors. These DCNNs have an extremely deep layer. The deployment of DCNNs in many real-world applications is largely hindered by their high computation cost [19]. During inference time, the intermediate responses of DCNN take a lot of time to calculate millions of parameters. As an example, YOLOv4 has 245 MB volume of parameters. Those parameters, along with network learning information, need to be stored on disk and loaded into memory while using YOLOv4 to detect objects. This process exerts a considerable resource burden on many platforms with limited memory and computing power.

Thus, in order to detect drones in real-time, we must reduce the number of parameters and computing operations. Many works have been proposed to compress DCNN [20,21,22]. Network slimming was proposed by Zhang Liu [19], which takes wide and large CNN as an input model and yields thin and compact models with comparable accuracy. SlimYOLOv3 was presented, which had fewer trainable parameters and floating-point operations (FLOPs) in comparison to original YOLOv3 by pruning convolutional channels [21]. These methods are referred to as pruning. The pruning network is identified as a standard and effective technique to remove unnecessary or unimportant convolutional channels from a DCNN to reduce its storage footprint and computational demands [23,24]. In this paper, we not only prune the convolutional channels, we also prune the layers. To the best of our knowledge, no study focused on pruned DCNN for real-time drone detection. 

Another problem about detecting drones is caused by the fact that the pixels of drones in the image only occupy an extremely small piece. This problem makes spotting drones difficult for a detector. Moreover, there is a significant gap in the CNNs’ performance between the detection of small and large objects. Therefore, in order to improve the accuracy of small drones and reduce the lost accuracy of the pruned model, we implemented a special augmentation for small object detection. The images containing small drones are picked up and then we copy-paste small drones multiple times. This augmentation can increase the number of small drones in each image and the diversity in the locations of small drones.

The rest of the paper is organized as follows. Section 2 introduces the related work about the object detectors based DCNN and small drone detection. Section 3 describes the materials and methods adopted in this paper, including the information about drone data, the pruning network, and small object augmentation technique. Section 4 presents and discusses the results obtained from the experimental analysis. In the end, Section 5 summarizes the main conclusions drawn from this study.

## 2. Related Work

Drone detection can be performed through radar, sound, video and radio frequency technologies as discussed above. In this paper, image processing is performed to monitor the presence of drones. Object detection methods are updated frequently in the field of computer vision. In this section, we introduce three excellent methods that have appeared in recent years. They are RetinaNet [25], FCOS [18] and YOLOv4 [26].

- RetinaNet: RetinaNet is a one-stage object detector that can address the problem of class imbalance by using a loss function called focal loss. Class imbalance is the situation in which the number of background instances is considerably larger than that of the target object instances. Thus, class imbalance wastes the network’s attention on the background, and the features of the target object cannot be learned sufficiently. Focal loss enables the network to focus on hard examples of the object of interest and prevents a large number of background examples from inhibiting method training.
- FCOS: Like RetinaNet, FCOS is a fully convolutional one-stage object detector to solve object detection in a per-pixel prediction, analog to semantic segmentation [18]. FCOS disregards the predefined anchor boxes, which play an important role in all state-of-the-art object detectors, such as Faster RCNN [27], RetinaNet, YOLOv4 and single shot multi-box detector [28]. Instead of anchor boxes, FCOS predicts a 4D vector (*l*, *t*, *r*, *b*) that encodes the location of a bounding box at each foreground pixel. Given its fully convolutional networks [29], FCOS can eliminate the fixed size of the input image. The network architecture of FCOS is composed of a backbone, a feature pyramid, and center-ness. ResNet-50 can be used as FCOS’s backbone, and the same hyper-parameters as those in RetinaNet are used.
- YOLOv4: Similar to RetinaNet, YOLOv4 is also a one-stage object detector. YOLOv4 is an improved version of YOLOv3. The YOLOv4’s backbone is CSPDarknet53 and the detector head is as same as YOLOv3 [30]. YOLOv3 predicts bounding boxes at three different scales to more accurately match objects of varying sizes. YOLOv3 extracts features from scales by using a concept similar to a feature pyramid network. For its backbone, YOLOv3 uses Darknet-53 because it provides high accuracy and requires fewer operations compared with other architectures. Darknet-53 uses successive 3 × 3 and 1 × 1 convolutional layers and several shortcut connections. Backbone networks extract features and generate three feature maps with different scales. The feature maps are divided into *S × S* grids. For each grid, YOLOv3 predicts the offset of bounding boxes, an objectness score, and class probabilities. YOLOv3 predicts an objectness score for each bounding box by using logistic regression. Compared with YOLOv3, YOLOv4 also adopts SPP and PAN structures to improve the ability of feature extraction. Meanwhile, probabilities are predicted for each class contained in the dataset. In this study, the number of classes is one, i.e., UAV.

Although DCNNs have strong representation power, they require more computing and storage resources. For example, YOLOv4 has more than 60 million parameters when inferencing an image with a resolution of 416 × 416. For the task of detecting a swiftly flying drone, such a huge calculation amount is not conducive to real-time detection. Resource-constrained platforms, such as embedded and internet of things devices, will not be affordable. To address this issue, many studies have proposed compressing large CNNs or directly learning more efficient CNN models for fast inference. Low-rank decomposition uses singular value decomposition to approximates weight matrix in neural networks [30]. In [10], they used a low-rank-based method to generate the drone proposal. Weight pruning is proposed to prune the unimportant connections with small weights in neural networks [31]. In [21], they pruned the convolutional channels of YOLOv3 to get the SlimYOLOv3 with fewer trainable parameters in comparison of original YOLOv3. However, SlimYOLOv3 is limited to pruning the channel, the layer cannot be pruned. In this paper, we not only improve the method of channel pruning in SlimYOLOv3 to prune the channel of the convolutional layer, but also prune the whole convolutional layer to obtain the slim and shallow models.

For detecting small drones, authors in [11] proposed low-rank and spare matrix that were utilized to decomposite the image and achieve the flying small drones by separate target images. In [10], another low-rank-based model was adopted to obtain the drone object proposals. These methods based low rank can detect the small drones, but they are not good at detecting the large drones. On the other hand, these DCNN detectors are good at detecting large drones. However, they struggle with the detection of small drones. Therefore, we propose small object augmentation to improve the ability of the detection of small drones. The main contributions of this paper are twofold:

- The integration of the advanced object detectors and pruned YOLOv4 which can detect drone in real-time;
- Our detector can be not only good at detecting large drones but also small drones.

## 3. Small Drones Detection

YOLOv4 can exhibit significant performance in image identification that attributes to deep and large network framework and massive data. In this section, we introduce the images that we collected for training and testing and the videos that we recorded for testing. Then, the pruned method is detailed. The special data augmentation for the small object will be presented.

### 3.1. Data Acquisition

**Figure 1:**
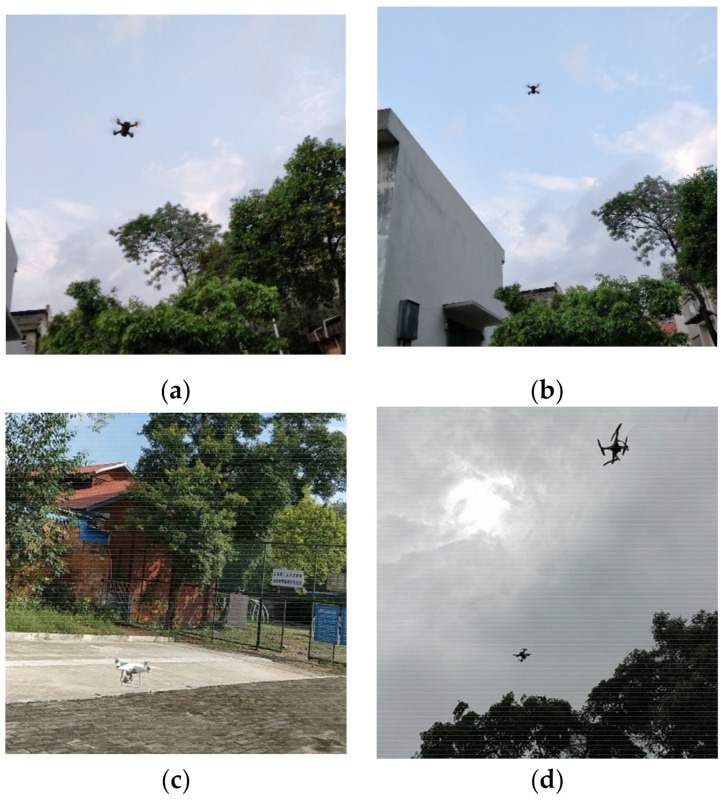
Examples from the datasets: images (**a**) and (**b**) contain DJI spark; image (**c**) contains DJI phantom and image (**d**) contains both DJI spark and phantom.

In total, ten thousand images of drones were acquired by the camera of a Oneplus phone that was used to take pictures of a small drone, DJI spark, and a big drone, DJI phantom. Among them, 4000 pictures only contain spark, 4000 pictures contain phantom, and the remaining 2000 pictures contain spark and phantom. Then, all images were randomly divided into two sets. The first set, called the training set, contained 8000 images. The remaining 2000 images comprised the testing set. Samples of drone images are shown in Figure 1. We took drone pictures at different angles and distances. Each image was annotated using a professional software called LabelMe, and the corresponding XML file that contained the coordinates of the top left and bottom right corners of the drone was generated. 

### 3.2. Pruned YOLOv4 

**Figure 2:**
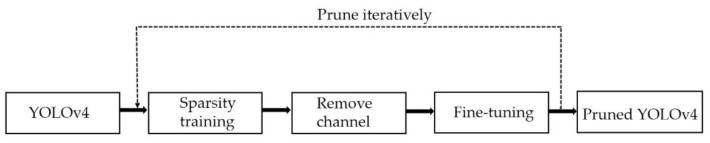
Block diagram of the proposed framework.

Among these three object detectors based on DCNN, YOLOv3 has many variants, of which SlimYOLOv3 is the variant of pruned YOLOv3 as a promising solution for real-time object detection on drones. Similarly, we prune YOLOv4 in this paper, and the procedure of pruning YOLOv4 is illustrated in Figure 2. 

The first step in pruning, which is also the most important step, is sparsity training. Sparsity training describes the number of less important channels that maybe be removed afterward. To implement channel pruning, an indicator is assigned to denote the importance of each channel. This indicator is called the scaling factor in SlimYOLOv3. Batch norm (BN) layers, which accelerate convergence and improve generalization, follow each convolutional layer in YOLOv4. A BN layer normalizes convolutional features by using mini-batch statistics, which can be expressed as Equation (1):

(1) $$y=\gamma \times \frac{x-\bar{x}}{\sqrt{\sigma^{2}+\varepsilon }}+\beta$$

where $\bar{x}$ and $\sigma^{2}$ are mean and variance of input feature x, γ and β denote trainable scale factor and bias, respectively. Thus, SlimYOLOv3 adopts the trainable scale factors in the BN layers as indicators and performs channel-wise sparsity training by imposing L1 regularization on γ. γ is used to discriminate important channels from unimportant channels effectively. The final loss of sparsity training is formulated as Equation (2):

(2) $$J(\gamma )=L{\left(\gamma \right)}_{yolo}+\alpha {\left\|\gamma \right\|}_{1}$$

where ${\left\|\gamma \right\|}_{1}$ denotes L1-norm, $L{\left(\gamma \right)}_{yolo}$ denotes the loss of YOLOv4, and α denotes penalty factor that balances the two loss terms. When α=0, there is no L1-norm. Then, Equation (2) uses Taylor’s formula to expand at $\gamma^{*}$:

(3) $$J(\gamma )=L{\left(\gamma^{*}\right)}_{yolo}+\frac{1}{2}\left(\gamma -\gamma^{*}\right)*H*{\left(\gamma -\gamma^{*}\right)}^{T}$$

where H is Hessian matrix. Assuming that γ in the parameters are independent of each other, then the Hessian matrix can become a diagonal matrix:

(4) $$H=\mathrm{diag}\left(H_{1,1},H_{2,2},\ldots H_{n,n}\right)$$

Then, Equation (2) can be formulated as Equation (5):

(5) $$J(\gamma )=L{\left(\gamma_{}^{*}\right)}_{yolo}+\sum_{i}\left[\frac{1}{2}H_{i,i}{\left(\gamma_{i}-\gamma_{i}^{*}\right)}^{2}+\alpha \left|\gamma_{i}\right|\right]$$

Coupled with the assumption of mutual independence, then we can get Equation (6):

(6) $$J(\gamma_{i})=L{\left(\gamma_{i}\right)}_{yolo}+\frac{1}{2}H_{i,i}{\left(\gamma_{i}-\gamma_{i}^{*}\right)}^{2}+\alpha \left|\gamma_{i}\right|$$

Derivative of the above formula, then the Equation (7) can be obtained:

(7) $$H_{i,i}\left(w_{i}-w_{i}^{*}\right)+\alpha *\mathrm{sign}\left(w_{i}^{*}\right)=0$$

where sign function can be described as Equation (8):

(8) $$sign(x)=\left\{\begin{array}{c} \begin{array}{l} 1,x>0\\ 0,x=0 \end{array}\\ -1,x<0 \end{array}\right.$$

Then, we can get $\gamma_{i}$:

(9) $$\gamma_{i}=\left\{\begin{array}{ll} 0 & \left|\gamma_{i}^{*}\right|\leq \frac{\alpha }{H_{i,i}}\\ \mathrm{sign}\left(\gamma_{i}^{*}\right)\left(\left|\gamma_{i}^{*}\right|-\frac{\alpha }{H_{i,i}}\right) & \left|\gamma_{i}^{*}\right|>\frac{\alpha }{H_{i,i}} \end{array}\right.$$

When more and more values of γ are close to 0, the goal of sparse BN weights is achieved.

**Figure 3:**
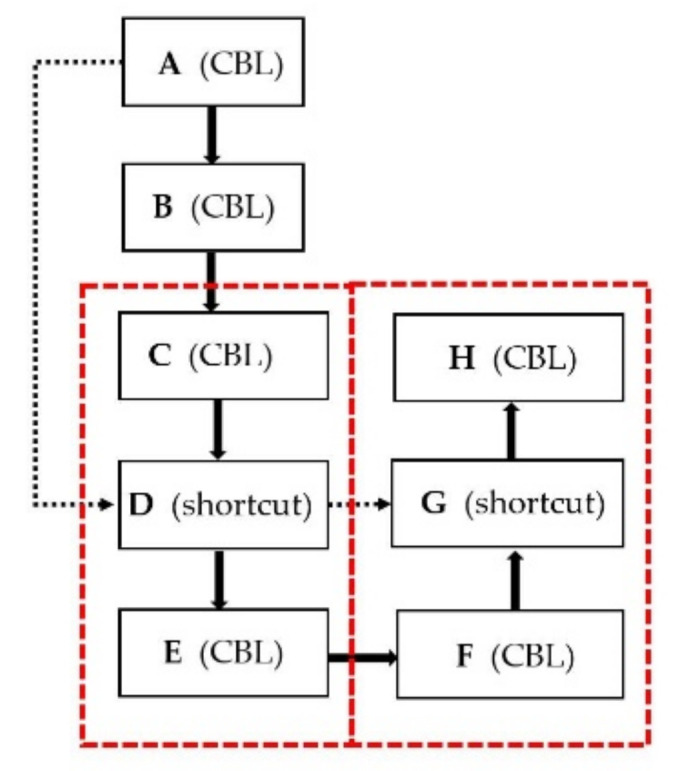
Shortcut layer structure of YOLOv4.

After sparsity training, γ has been attached to determine how a feature convolutional channel is important. The pruning ratio is set to remove the relatively unimportant channel with a scaling factor lower than the product of the pruning ratio and the scaling factor. After pruning these channels, the dimension of the weight of the layers connected to the pruned layer should be adjusted, particularly the shortcut layer [21]. To match the feature channels of each layer connected by shortcut layer, the author of SlimYOLOv3 iterated through the pruning masks of all connected layers and performed OR operation on these masks to generate a final pruning mask for these connected layers [21]. Nearly each layer of YOLOv4 is composed of a convolutional layer, a BN layer and a rectified linear unit activation layer (CBL). The shortcut layer structure is shown in Figure 3. YOLOv4 has 23 shortcut layers in total. For example, both A layer and C layer are the input of the shortcut layer D. To ensure the integrity of the YOLOv4 structure, the reserved channels of A layer and C layer must be consistent. If A layer retains A1 and A2 channels, C layer retains C1 and C3 channels and layer F retains F3 and F4 channels, then after the OR operation, layers A,C,D,F and G will retain 1,2,3 and 4 channels. This efficiency of pruning the shortcut layer is too low. In this paper, in order to achieve a greater degree of channel pruning, we use other operation to prune the shortcut layer. At first, we refer to the first layer in all shortcut related layers as a leader. Then, other shortcut related layers reserve the channels as same as the leader’s. In other words, layer A is the leader, then layers A, C, D, F and G will retain 1 and 2 channels.

Although we can increase the intensity of channel pruning, SlimYOLOv3 is limited to pruning channels and it does not prune layers. For our task of detection drones, YOLOv4 with 159 convolutional layers may be too complicated. In this study, the layer of YOLOv4 is pruned too. Pruning each shortcut layer will cause three layers, which are in the red dotted box in Figure 3, to be removed. The mean value of γ of each shortcut layer is evaluated. For example, the mean value of γ of D layer is an indicator of B, C and D. If the shortcut layer D is being pruned, then C, D and E are being pruned. Layer pruning is done after channel pruning. Certainly, only the shortcut module in the backbone is considered in this study. Therefore, we can prune the layer and the channel. Correspondingly, the approach of pruned YOLOv4 can be presented based on all above discussed modules and outlined as Algorithm 1.
**Algorithm 1.** Approach of pruning channel and layer in YOLOv4 **Input:** N layers and M shortcut layers of YOLOv4, channel pruning rate α and layer pruning t
**Output:** The remaining layers after pruning Sparsity training N layers and M shortcut layers and get $\gamma_{k}^{i}$ of the k-th channel of i-th layer Sort $\gamma_{k}^{i}$ of N layers and M shortcut layers from small to large and then get array W Threshold $t=W\left[int\left(\alpha \cdot len\left(W\right)\right)\right]$ **for** i=1 to N
**do**
if $\gamma_{k=\left\{1,2,\ldots \right\}}^{i}<t$ Remove these channels $k=\left\{1,2,\ldots \right\}$ of i-th layer
**end for** A~F is shown as Figure 3. $A^{i}$ is the A layer of i-th shortcut layer structure. **for** i=1 to M do   if $\gamma_{k=\left\{1,2,\ldots \right\}}^{i}<t$ Mark $k=\left\{1,2,\ldots \right\}$ which is the index of channels of $A^{i}$ layer  **for** $j=A^{i}$ to [$A^{i}$,$\;C^{i}$,$\;F^{i}$] **do**  Remove $k=\left\{1,2,\ldots \right\}$ channels of j layer **end for** **end for** Evalute the mean value $m_{s=\left\{1,2,\ldots ,23\right\}}$ of $\gamma_{k=\left\{1,2,\ldots \right\}}^{i}$ for each M shortcut layers, then sort m from small to large **for** i=1 to t **do** Get the index of shortcut layer $s=m_{s}\left[i\right]$ Remove $C^{s},D^{s}$ and $E^{s}$ layers **end for**

### 3.3. Small Object Augmentation 

The drone is difficult to detect because it is not only moving swiftly but it also becomes smaller as it flies higher. To address this problem, an augmentation method for small object detection is applied. The small object is defined in Table 1 in the case of the Microsoft Common Objects in Context (MS COCO) dataset [32]. According to statistics, there are 7928 images with 9388 small objects in whole dataset. It can be seen that the probability of small objects in this dataset is extremely high.

**Figure 4:**
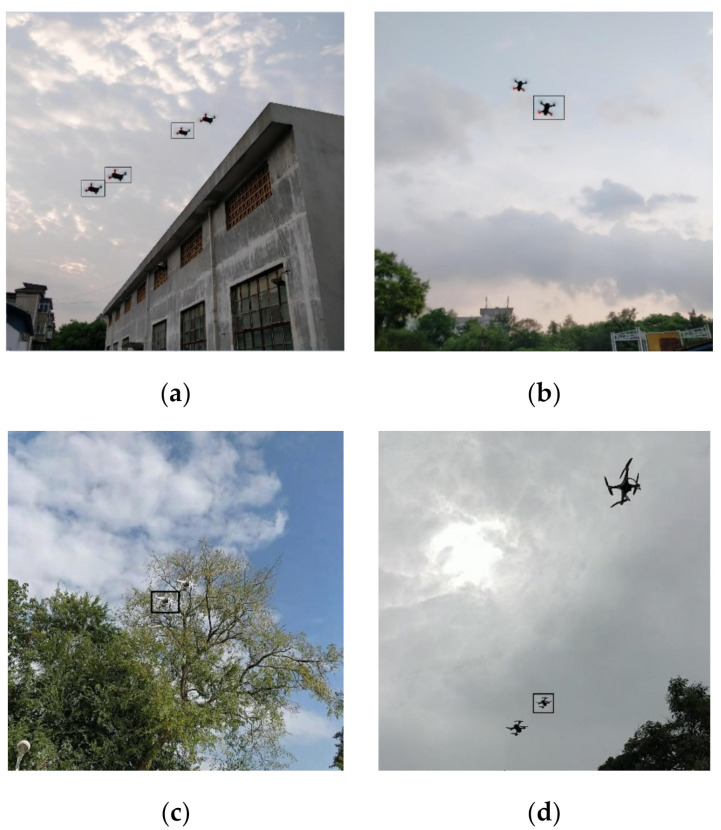
Examples of artificial augmentation by copy-pasting the small drone in the images containing small drone. The drones in black boxes are the copy-pasted drones. (**a**) a spark with three copy-pasted sparks; (**b**) a spark with a copy-pasted spark; (**c**) a phantom with a copy-pasted phantom; and (**d**) a spark and a phantom with a copy-pasted spark.

The small object cannot be detected easily due to the fact that small objects do not appear enough even within each image containing them. This issue can be tackled by copy-pasting small objects multiple times in each image containing small objects. As shown in Figure 4, the pasted drones should not overlap with any existing object. The size of a pasted drone can be scaled by changing ±0.2. In Figure 4, all images contain a small drone, and their augmentations are shown in black boxes. Either DJI spark or phantom has a possible case of being a small object. The number of matched anchors increases by increasing the number of small objects in each image. This small drone augmentation method can drive a model to focus more on small drones. Moreover, it can improve the contribution of small objects to the computation of the loss function during the training of the detector model.

## 4. Experimental Results

This section presents the experimental results. Firstly, we explore which DCNN detector can achieve better performance and be more suitable for pruning. Secondly, we apply pruning channel and layer to the detector selected in Section 4.1. At last, for small drone detection, the special augmentation is discussed. 

### 4.1. Result of Four DCNN-Based Model

The mean average precision (mAP) is the primary evaluation matrix in the detection challenge. In this paper, we also use mAP as the evaluation of the performance of each method. In general, mAP is defined as the mean average of ratios of true positives to all positives and for all recall values [33]. For the object detection, a detector needs to both locate and correctly classify, a correct classification is only counted as a true positive detection if the predicted mask or bounding box has an intersection-over-union (IoU) higher than 0.5. Following well-known competitions in object detection [16], a correct detection (True Position, TP) is considered for IoU ≥ 0.5, and a wrong detection (False Positive, FP) for IoU < 0.5. A False Negative (FN) is assigned when no corresponding ground truth is detection. Precision and recall are estimated using Equations (10) and (11), respectively. In our task, a detector only needs to classify whether the located object is a drone.

(10) $$P=\frac{TP}{TP+FP}$$

(11) $$R=\frac{TP}{TP+FN}$$

In addition to mAP, F1-score is the harmonic average of precision and recall as Equation (12).

(12) $$F_{1}=2*\frac{P*R}{P+R}$$

where P and R are obtained by Equations (4) and (5), respectively. F1-score can more scientifically indicate the validity of classification.

RetinaNet have been reproduced by the developer of FCOS. In order to enhance the comparability of the experiment, we utilize the FCOS code to compare the performance of both FCOS and RetinaNet. FCOS is tested based on ResNet-50 and ResNet-101. The performance of FCOS is shown in Table 2. The better performance is achieved by ResNet-101. Nevertheless, the mAP floats under different parameters, but there is no large deviation. The model with the backbone of ResNet-50 is more suitable for our task because the model with backbone of ResNet-101 pays the cost of adding a lot of calculations but mAP does not make a considerable improvement. The mAP value of RetinaNet is also shown in Table 2. A great performance is attained by the RetinaNet. The performance of other detectors is also presented in Table 2.

**Figure 5:**
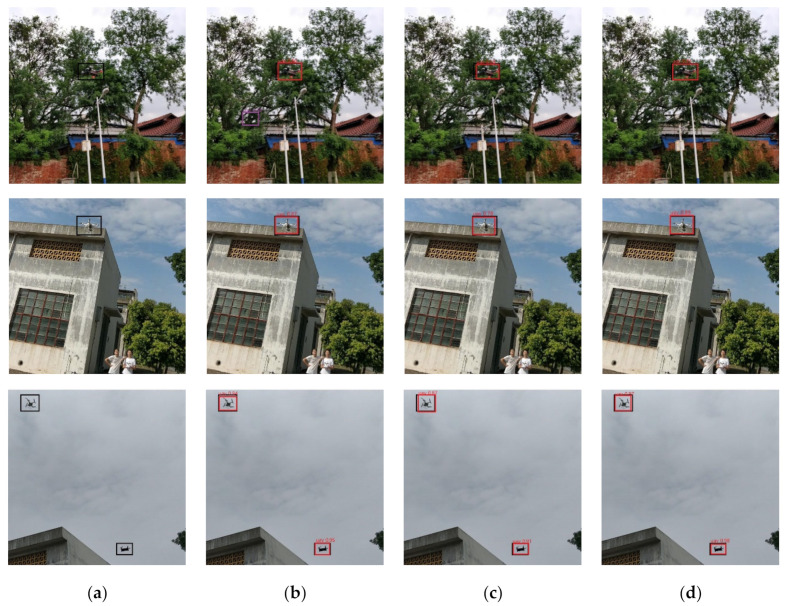
Examples of detection results: (**a**) ground truth; (**b**) FCOS with ResNet-50; (**c**) RetinaNet; and (**d**) YOLOv4. The ground truth box, truth prediction box and false prediction box are black, red and purple, respectively.

In this paper, YOLOv3 and YOLOv4 adopt the input size of 416. YOLOv3 can achieve comparable performance with other detectors. YOLOv3 has been widely used in the industry because of its excellent trade-off between speed and accuracy. YOLOv4 has the same potential as YOLOv3. In this task, YOLOv4’s performance in all aspects is better than other algorithms. The mAP of YOLOv4 can achieve 93.6%. The precision and recall of YOLOv4 also obtain excellent performance. The examples of detection results are shown in Figure 5. The first column shows the ground truth images while the three columns on the right present the results produced by the three detection methods, namely FCOS with ResNet-50, RetinaNet and YOLOv4. The threshold of the test phase is set to 0.3. All the results are fine, except for the false prediction box in FCOS. The possible reason is that precision is too low. Especially in such a complex background, it is easy to appear the false prediction box. The In the next section, we prune YOLOv4 to obtain a faster detector.

### 4.2. Result of Pruned YOLOv4

In this paper, we use YOLOv4 as our baseline model. Before YOLOv4 can be pruned, it needs sparse training. In order to prove the importance of sparse training, we carry out the experiment of pruning channel without sparse training as shown in Table 3. The mAP of the pruned model drops rapidly if spare training has not been done. Sparsity training is able to effectively reduce the scaling factors and thus make the feature channels of convolutional layers [21].

**Figure 6:**
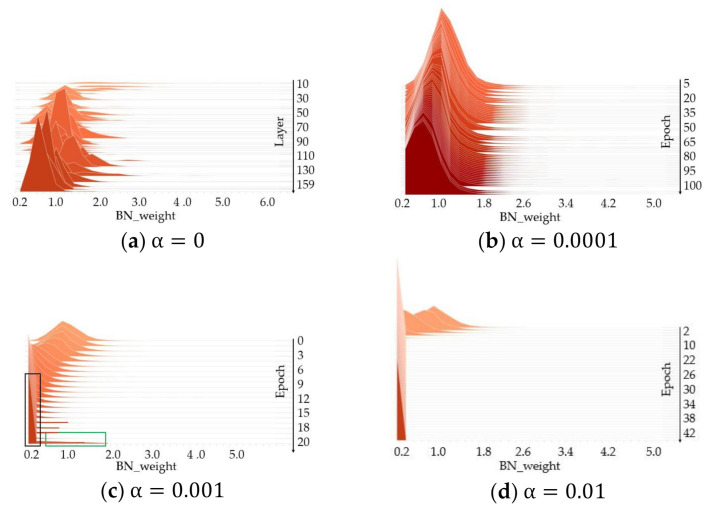
Histogram statistics of scaling factors in all BN layers with two different values of α including 0.001, 0.005 and 0.01.

Before training, we stack the distribution of weights for layers of YOLOv4, which has 159 layers, as shown in Figure 6a. Most of the BN weights move from 2.0 to around 1.0 as the number of layers increases. The degree of sparsity is determined by the scale factor and the number of epochs together. During the sparsity training, we compute the histogram of the absolute value of weights in all BN layers of YOLOv4 and stack them in one figure to observe the trend. As shown in Figure 6b, we adopt the weaker scale factor α=0.0001 to sparse the weight. The channel whose BN weight is close to zero is unimportant. The more channels are unimportant, the more channels we can prune. We can observe that the weight does not clearly tend to 0 from Figure 6b. As shown in Figure 6c, the weight in black box is pruned preferentially over other weight in green box. Additionally, the weight in green box is considered to be the more important weight, which is able to help improve accuracy in terms of fine-tuning. Sparsity training with a larger scale factor, i.e., α=0.01, makes the BN weight decay so aggressively that the pruned model will have a higher training difficulty and then fail with underfitting. Thus, in our experiments, we use the YOLOv4 model trained with penalty scale α=0.001 to perform channel and layer pruning.

We evaluate all the pruned models on the basis of the following metrics: (1) mAP; (2) model volume, which is the size of the weight file; and (3) frames per second (FPS) with GPU, which is Tesla P100 in our work. Among them, FPS is the indicator of detection speed. When we set the pruned channel ratio, we should also set the kept channel ratio to avoid the likelihood of pruning all the channels in a layer. We compare the detection performance of all the pruned models in Table 4. We can observe that channel pruning can cause the volume of mode to decrease rapidly, particularly when the pruned channel ratio is 0.5, the volume of a pruned model ranges from 245.8 MB to 90.8 MB.

**Figure 7:**
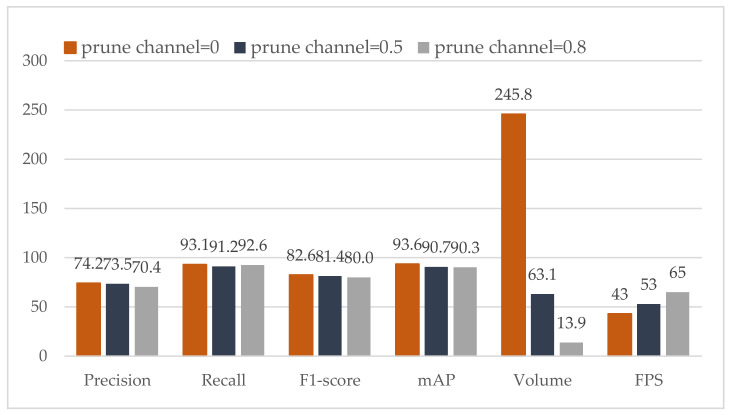
Performance comparison of YOLOv4 and our pruned channel models.

The evaluation of the pruned channel model is shown in Figure 7. We compare the performance of the prune rates of 0.5 and 0.8. Notably, when the prune rate or prune layer is 0, it means YOLOv4. As can be seen from Figure 7, precision, recall, F1-score and mAP all have a slight drop. The volume of these models drops significantly. More importantly, FPS is improved considerably. When the prune rate is equal to 0.8, FPS is almost increased by 50% with the same level performance as YOLOv4.

**Figure 8:**
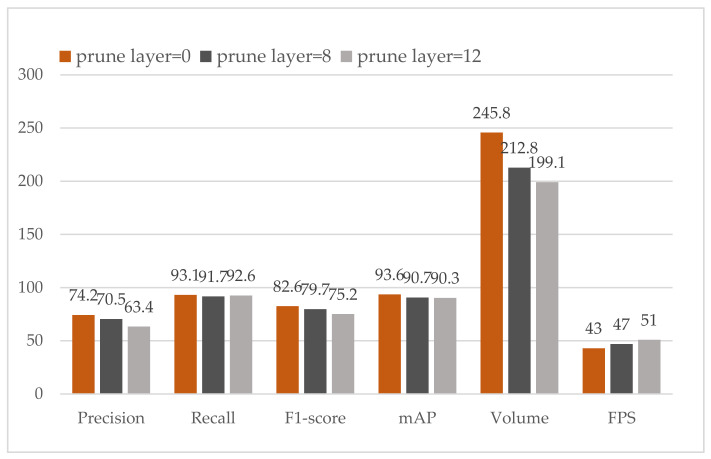
Performance comparison of YOLOv4 and our pruned layer models.

The performance of the pruned shortcut layer is illustrated in Figure 8. The recall and mAP have a slight drop. However, the precision declines as the number of prune layers increases. More notably, although volume does not fall as sharply as prune layer, FPS develops a comparable improvement. We can infer that prune layer can improve FPS even if it does not significantly reduce the volume of models.

Furthermore, we can combine the pruned layer and the pruned channel to gain a simpler and more effective model. As shown in Table 4, a pruned model with a prune channel ratio of 0.8 and a prune layer of 8 has an AP of 90.5 and a volume of 15.1 MB. Additionally, its FPS is improved by 60% while its performance of mAP achieves a comparable with YOLOv4. We use this model as our pruned-YOLOv4. Under the other settings of channel prune, layer prune and keep channel, FPS has different degrees of improvement.

**Figure 9:**
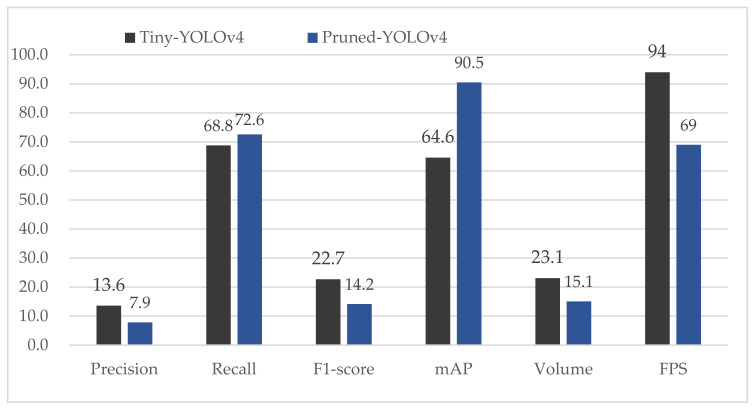
Performance comparison of the tiny-YOLOv4 and our pruned-YOLOv4 model.

In order to further demonstrate the effectiveness of our pruned model, we carry out one more comparative experiment. The tiny-YOLOv4 is an excessively simplified version of YOLOv4. The tiny-YOLOv4 only has 27 layers and a volume of 23.1 MB. We compare tiny-YOLOv4 and our pruned-YOLOv4 model, as shown in Figure 9. The tiny-YOLOv4 has a slight advantage in precision and F1-score. However, our pruned-YOLOv4 model has a strong advantage over tiny-YOLOv4 in mAP. Due to having less layers, the tiny-YOLOv4 outperforms on FPS. However, an FPS of 69 is not terrible in our task. Therefore, it can be concluded that our pruned model is able to effectively improve the detection speed with slight accuracy loss.

### 4.3. Result of Data with Small Object Augmentation

**Figure 10:**
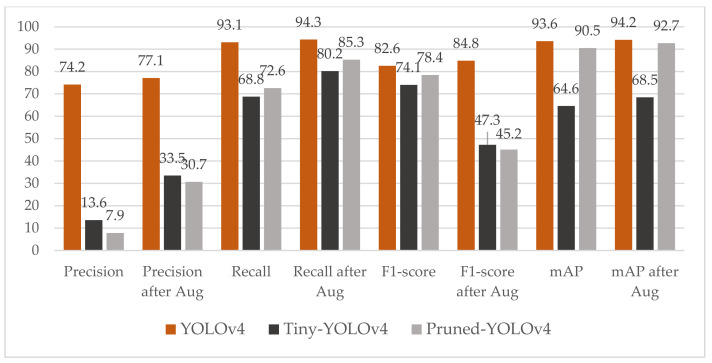
Performance comparison of the YOLOv4, tiny-YOLOv4 and our pruned-YOLOv4models.

The drawbacks of the pruned models are obvious: the value of precision, recall and F1-score have notable loss. For example, the value of precision drops from 74.2% to 7.9% in the first term of Figure 10. The lower the precision reveals that there are the more false detection boxes. Likewise, the value of recall drops from 93.1% to 72.6% in the third term of Figure 10. The lower recall demonstrates that the probability of missed detector of drones increases. The pruned model also results in degraded performance of mAP.

We infer the main reason for these problems is that a large number of small objects are difficult to be detected by the pruned-YOLOv4. Therefore, we implemented the small object augmentation to further improve the accuracy of detecting small drones. This augmentation method can only be implemented in our training dataset. We select a small drone from an image and then copy and pasted it multiple times in random locations. The augmented images replace the original ones and are stored in the training dataset. After augmentation, the detection ability of small drones is dramatically improved. All the terms of performance are improved by varying magnitudes. As shown in Figure 10, the precision of the pruned-YOLOv4 increases by 3 times after augmentation. Additionally, the recall of the pruned-YOLOv4 increases from 30.7% to 72.6%. Not only the pruned-YOLOv4, the tiny-YOLOv4 has also been similarly improved. The YOLOv4’s improvements in all aspects of performance are negligible. We hypothesize this is for the reason that YOLOv4 itself has a strong ability to detect the small objects. Meanwhile, the tiny-YOLOv4 and the pruned-YOLOv4 lose the ability of detection of the small objects due to the reduction of layers and channels.

**Figure 11:**
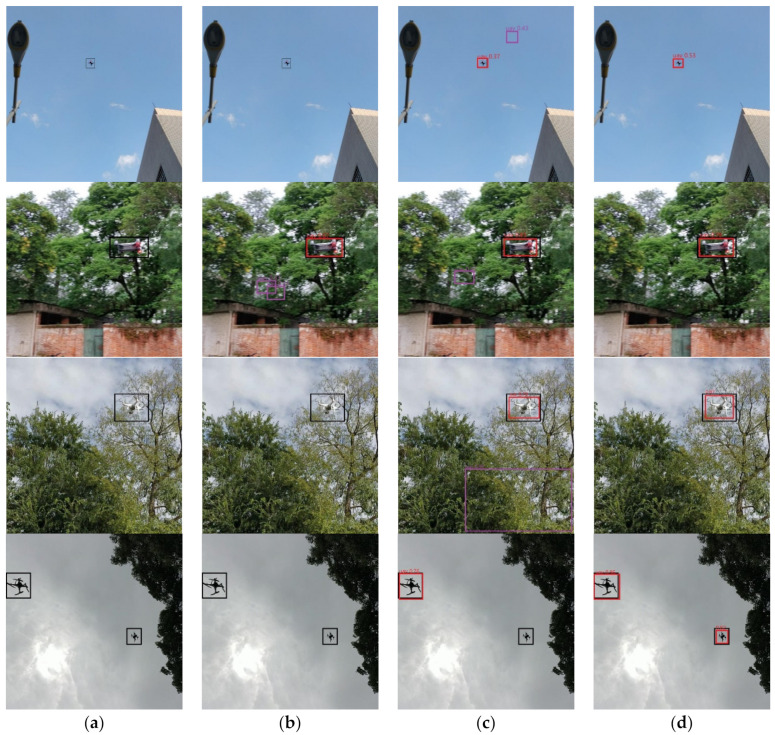
Examples of detection results: (**a**) ground truth; (**b**) tiny-YOLOv4; (**c**) pruned-YOLOv4; and (**d**) pruned-YOLOv4 after augmentation. The ground truth box, truth prediction box and false prediction box are black, red and purple, respectively.

In the comparison between the tiny-YOLOv4 and the pruned-YOLOv4, we still tend to choose the pruned-YOLOv4. They achieved similar performance in the terms of precision, recall and F1-score. However, the mAP of the pruned-YOLOv4 is 24.2% higher than the tiny-YOLOv4 after augmentation. This huge gap prompts us to choose the pruned-YOLOv4 instead of the tiny-YOLOv4. The examples of detection results are as shown in Figure 11. The second column shows the prediction results of the tiny-YOLOv4. Many drones are not detected. In the third column, only one spark is not detected in the last row, but lots of false boxes appear. In the last column, these mistakes are corrected. From these results, we infer that our pruned-YOLOv4 is a more suitable and reliable detector for the detection of drones by adopting pruning and small object augmentation.

## 5. Conclusions

In this paper, we propose an approach for the detection of small drones based on CNN. Four state-of-the-art CNN detection methods are tested: RetinaNet, FCOS, YOLOv3 and YOLOv4. These four methods achieve 90.3%, 90.5%, 89.1% and 93.6% mAP, respectively. YOLOv4 is our baseline model, with a volume of 245.8 MB and an FPS of 43. Additionally, we prune the convolutional channel and the shortcut layer of YOLOv4 with different parameters to obtain thinner and shallower models. Among these models, a pruned YOLOv4 model with 0.8 channel prune rate and 24 layers prune is as our pruned-YOLOv4, which can achieve 90.5% mAP, 69 FPS and 15.1 MB volume. That means our pruned-YOLOv4’s processing speed is increased by 60.4% with compromising a small amount of accuracy. We also implement an experiment to compare the tiny-YOLOv4 and our pruned-YOLOv4. Considering the trade-off between speed and accuracy, we still chose pruned-YOLOv4 as the detector.

Furthermore, we carry out small object augmentation to enhance the detection capability for small drones and compensate for accuracy loss. All the models are improved by different magnitudes. Although YOLOv4 is not greatly improved, the tiny-YOLOv4 and the pruned-YOLOv4 are greatly improved. The precision and recall of the pruned-YOLOv4 almost increases by 22.8% and 12.7%, respectively. These results show the pruned-YOLOv4 with small object augmentation has great advances for detecting small drones. In the future, we plan to further improve the loss accuracy due to pruning, and deploy the pruned model in embedded devices.

## Figures and Tables

**Table 1:** Definitions of the small, medium, and large object in MS COCO.

Object	Min Rectangle Area	Max Rectangle Area
Small Object	0 × 0	32 × 32
Medium Object	32 × 32	96 × 96
Large Object	96 × 96	∞ × ∞

**Table 2:** Performance of FCOS detector based on ResNet-50 and ResNet-101, Retinanet, YOLOv3 and YOLOv4.

Model	Precision	Recall	F1-Score	mAP
ResNet-50	12.9	94.9	22.7	85.5
ResNet-101	26.7	78.6	39.9	90.3
RetinaNet	68.5	91.7	78.4	90.5
YOLOv3	61.7	91.5	73.7	89.1
YOLOv4	74.2	93.1	82.6	93.6

**Table 3:** Performance of pruning without sparse training.

Pruned Ratio	mAP	Parameters(M)	FPS
0	93.6	63.9	43
0.10	91.2	52.1	46
0.15	69.3	47.6	55
0.20	12.9	43.4	60
0.85	0.0	8.3	79

**Table 4:** Evaluation results of pruned models.

Channel Prune	Layer Prune	Keep Channel	mAP	Volume(MB)	FPS
0	0	1	93.6	245.8	43
0.5	0	0.01	90.8	63.1	53
0.8	0	0.01	86.3	13.9	65
0.9	0	0.01	64.1	6.61	79
0	8	1	90.7	212.8	47
0	12	1	90.3	199.1	51
0.5	12	0.1	90.8	52.9	64
0.8	8	0.1	90.5	15.1	69
0.8	8	0.01	83.6	10.9	71
0.8	12	0.1	78.4	9.9	75
0.9	8	0.01	66.5	7.4	77
0.9	8	0.1	68.3	7.9	76

## Data Availability

Not applicable.
